# Diurnal regulation of the floral scent emission by light and circadian rhythm in the *Phalaenopsis* orchids

**DOI:** 10.1186/s40529-017-0204-8

**Published:** 2017-11-15

**Authors:** Yu-Chen Chuang, Ming-Chi Lee, Ya-Lan Chang, Wen-Huei Chen, Hong-Hwa Chen

**Affiliations:** 1 Department of Life Sciences, National Cheng Kung University, Tainan, 701 Taiwan; 2 Orchid Research and Development Center, National Cheng Kung University, Tainan, 701 Taiwan

**Keywords:** Circadian clock, Floral scent, Light-regulated, Monoterpene, *Phalaenopsis*

## Abstract

**Background:**

*Phalaenopsis bellina* and its closely related species, *P. violacea*, emit linalool, geraniol and their derivatives as the predominant monoterpenes at the full-bloom stages. Geranyl diphosphate synthase (PbGDPS) is the key enzyme that converts precursors for monoterpene biosynthesis. Besides the monoterpenes being synthesized in concert with floral development stages, we noticed that the scent emission of *P. bellina* and *P. violacea* was detected mainly in the daytime.

**Results:**

The monoterpenes of *P. violacea* flowers displayed a diurnal emission pattern, which was regulated by an internal oscillator in the treatment of constant light. In contrast, constant dark diminished the scent emission levels, indicating that light also affects monoterpene emission in *P. violacea*. Further treating *P. violacea* with various light wavelengths showed that the monoterpene emission was greatest in white light condition. Other *Phalaenopsis* hybrids, including *P.* I-Hsin Venus ‘KHM2212’ and *P.* Meidarland Bellina Age ‘LM128’, responded differently to various light wavelengths but most of them still showed the highest scent emission under the whole spectra of light. A great number of light-responsive, HY5-interacting, and circadian-responsive elements was enriched on the promoters of both structural genes and transcription factors for monoterpene biosynthesis. Furthermore, several putative genes encoding components involved in light and circadian signaling pathways were also identified in the transcriptome of *P. bellina* flowers at consecutive stages (from the anthesis day to day 7 post anthesis).

**Conclusions:**

Taken together, both circadian clock and light factors had positive effects on orchid floral scent emission, and the regulation resided on the control of both structural genes and transcription factors for monoterpene biosynthesis.

## Background

Floral scent is an important factor to attract pollinators for reproduction. To achieve this, the floral scent is often developmentally and rhythmically regulated to be associated with the activity of corresponding pollinators (Muhlemann et al. 2014). As insect pollinators exhibit rhythmic activities (Saunders 1997), the flowers emit scent on specific timing in a day (Hoballah et al. 2005; Fenske and Imaizumi 2016). This diurnal/nocturnal emission is proposed to be regulated by a circadian clock.

The circadian clock implicates an endogenous oscillator so as to control its downstream physiological process rhythmically even under constant condition. The oscillator is established by synchronizing to the environment stimuli including light and temperature; the two factors change extremely during day/night cycle (Devlin and Kay 2000). The light receptors are responsible for inputting the light signal into the circadian clock (McClung 2006).

Light directly affects floral scent emission as well. Plants treated with various light intensities and light wavelengths show fluctuation changes of their volatiles. 2-Phenylethanol from petunia flowers is increased under red and far-red light treatments (Colquhoun et al. 2013; Abe et al. 2003). Chiloglottone production in *Chiloglotti*s orchids require UV-B light (Falara et al. 2012). The volatiles emitted from tea leaves are raised by blue light and red light (Fu et al. 2015).

*Phalaenopsis bellina* and its genetically as well as morphologically related species, *P. violacea* (Tsai 2003), are usually used in the breeding of scented cultivars. There are approximately 730 *Phalaenopsis* hybrids with *P. bellina* or *P. violacea* as parents registered in The Royal Horticultural Society. The floral scent profiles of *P. bellina* are dictated by monoterpenes, including linalool and geraniol and their derivatives (Hsiao et al. 2006). Geranyl diphosphate synthase of *P. bellina* (PbGDPS) is the key enzyme to supply geranyl diphosphate (GDP) for monoterpene biosynthesis. Its maximal expression is concomitant with the peak emission of monoterpenes at the full-bloom stage (day 5 post anthesis, D + 5) (Hsiao et al. 2008). Expression of *PbGDPS* and its downstream putative monoterpene synthases, TERPENE SYNTHASE 5 (*PbTPS5*) and *PbTPS10*, are regulated during flower development by several transcription factors (TFs), including PbbHLH4, PbbHLH6, PbbZIP4, PbERF1, PbERF9, and PbNAC1 (Chuang et al. unpublished).

We noticed that floral scent of *P. bellina* and *P. violacea* showed a pattern of diurnal emission in summer. The emission levels were reduced under cloudy and rainy days, even at full blooming stages, prompting us to investigate whether light or circadian rhythm plays an important role in the production of floral scent in orchids. In addition, the proposed components involved in the signal pathway were discussed as well.

## Methods

### Plant materials and growth condition

Two native species of *Phalaenopsis* orchids, *P. bellina* and *P. violacea* were obtained from Han-Lin Orchids (Tainan, Taiwan). Two commercial cultivars were also included in the analysis. *P.* I-Hsin Venus ‘KHM221’ (abbreviated as ‘KHM221’ in the following) is derived from a cross between ‘*P.* I-Hsin Viola Tris’ and ‘*P.* Dragon’s Gold’ from I-Hsin Biotechnology Co. (Chiayi, Taiwan). The other cultivar, *P.* Meidarland Bellina Age ‘LM128’ (abbreviated as ‘LM128’ in the following) is derived from a cross between ‘*P*. K. S. Happy Eagle’ and *P. bellina* from Meidarland Orchids (Tainan, Taiwan). The genetic constitution of both cultivars was downloaded from ORCHIDEYA.CA (http://www.orchideya.ca/) as follows: ‘KHM221’—*P. amboinensis* (25%), *P. equestris* (25%), *P. venosa* (18.75%), *P. violacea* (15.63%), *P. rimestadiana* (5.71%), *P. amabilis* (4.69%), *P. lueddemanniana* (3.13%), and *P. aphrodite* (2.1%). For ‘LM128’, the parenthood is the following—*P. bellina* (50%), *P. violacea* (24.22%), *P. venosa* (15.63%), *P. amboinensis* (3.91%), *P. lueddemanniana* (3.13%), and *P. micholitzii* (3.13%). Among these native *Phalaenopsis* orchids, several are scented *Phalaenopsis*, including *P. amboinensis*, *P. bellina*, *P. lueddemanniana*, *P. venosa*, and *P. violacea*. All *Phalaenopsis* plants were grown in the greenhouse at National Cheng Kung University (NCKU) under natural light around 100 μmol m^−2^ s^−1^ (LI-COR photometer, United States) and controlled day/night temperature 30 °C/28 °C with 80% humidity in summer.

### Light treatments

Plants were transferred to the growth chamber (F180 LED, Hi Point Corporation, Taiwan) on the day of anthesis (Dd). *P. violacea* was first analyzed by long day (16 h/8 h light/dark (L/D) or short day condition (8 h/16 h L/D). For analyzing whether floral scent emission is regulated by circadian rhythm or by light, six plants were treated with white light (16 h/8 h L/D) for 4 days (from Dd to D + 4), and then divided into two groups under the treatment of either constant light or constant dark for the following 4 days (from D + 5 to D + 8). Floral scent was collected from D + 4 to D + 8 during four periods of time (from 4 a.m. to 9 a.m., 10 a.m. to 4 p.m., 4 p.m. to 9 p.m., and 10 p.m. to 4 a.m.) each day.

To study the responses of floral scent emission to various light qualities, orchid plants were treated with various light sources for 16 h/8 h L/D at 27 °C with 60% humidity. Plants were domesticated for 3–5 days before harvest. LED of three-wavelengths were used, including blue light (central wavelength 450 nm) at 270 μmol m^−2^ s^−1^, red light (central wavelength 650 nm) at 30 μmol m^−2^ s^−1^ and far-red light at 5 μmol m^−2^ s^−1^. The scent release of *P. violacea* flowers in response to different light conditions was analyzed with triplicate biological replicates. Four individual seedling plants of ‘LM128’ were used and the replicate number for each condition was as the following: (a) No. 1 plant (*n* = 3 for white light (WL), *n* = 4 for blue light (BL), (b) No. 2 plant (*n* = 1 for WL, *n* = 4 for BL), (c) No. 3 plant (*n* = 3 for both WL and BL), and (d) No. 4 plant (*n* = 3 for WL, *n* = 1 for BL) (Fig. 3). For statistical analysis between two groups, assuming equal variance, one-tailed Student’s *t* test was performed (*p* < 0.1). For ‘KHM221’ plants, 50 μmol m^−2^ s^−1^ white, blue, and red light conditions, and darkness were applied with duplicates.

### Solid-phase extraction and GC–MS analysis

Floral scent was collected from plants by using solid-phase extraction system. The flower on the plants was placed in a plastic bag (8 × 8 × 5 cm^3^) inserted with a silicone tube connecting the solid phase extraction columns (DSC-Si and DCS-18, Supelco, United States) using air pumps at 350 mmHg (Rocker, Taiwan). The volatiles were then eluted with the addition of hexane and identified by using (GC–MS; QP2010, SHIMADXU, Shimadzu Co, Tokyo, Japan) at the NCKU Instrument Center as previously described (Hsiao et al. 2006).

### Isolation of upstream regulatory fragments and *cis*-element prediction

Upstream regulatory fragments of three monoterpene biosynthesis genes, including *PbGDPS*, *PbTPS5*, and *PbTPS10*, were isolated from *P. bellina* genomic DNA by use of the Universal GenomeWalker Kit (Clontech, USA) as previously described (Hsu et al. 2014). In addition, we also isolated 1.5-kb upstream regulatory fragments of the identified six TFs involved in the regulation of floral monoterpene biosynthesis in *P. bellina* (Chuang et al. unpublished) from *P. equestris* genomic sequences (Cai et al. 2015). The *cis*-elements related to light signaling and circadian clock on the upstream regulatory fragments of both monoterpene biosynthesis genes and TFs were predicted by using PlantPAN (Chow et al. 2015). The predicted results with 100% similar score were accepted.

### Identification of genes involved in light and circadian clock signaling pathways

The *P. bellina* transcriptomic data of four floral development stages (Dd, D + 3, D + 5 and D + 7) were constructed, analyzed and annotated as described (Chuang et al. unpublished). The homologous genes of the *Arabidopsis* factors involved in light and circadian clock signaling pathways were identified in the *P. bellina* transcriptome according to Nr annotation. These included PHYTOCHROME A (PHYA), PHYTOCHROME B (PHYB), CRYPTOCHROME 1 (CRY1), CRYPTOCHROME 2 (CRY2), PHOTOTROPIN 1 (PHOT1), PHOT2, CONSTITUTIVE PHOTOMORPHOGENIC 1 (COP1), SUPPRESSOR OF PHYTOCHROME A1 (SPA1), PHYTOCHROME AND FLOWERING TIME 1 (PFT1), PHYTOCHROME-INTERACTING FACTOR 3 (PIF3), PIF7, HY5 (LONG HYPOCOTYL5), HY5-HOMOLOG (HYH), LATE ELONGATED HYPOCOTYL (LHY), CIRCADIAN CLOCK-ASSOCIATED 1 (CCA1), TIMING OF CAB EXPRESSION 1 (TOC1), and GIGANTEA (GI). The expression level of each individual gene in the transcriptomic data was determined by the log2 of fragments per kilobyte per million reads (FPKM) values. PHOT2, PFT1, PIF7, and TOC1 were excluded for final analysis since little or no expression for the four genes was detected.

## Results

### Effects of light on *P. violacea* scent emission

**Fig. 1:**
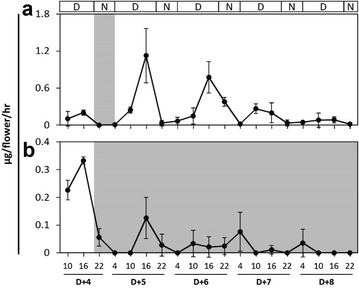
Emission of monoterpenes from *P. violacea* flowers exposed to different light/dark conditions. **a** Flowers were exposed in 16 h/8 h L/D condition for 5 days and followed by constant light (~ 100 μmol m^−2^ s^−1^) for 4 days. **b** Flowers were exposed in 16 h/8 h L/D condition for 5 days and followed by constant dark for 4 days. Floral scent was collected by applying solid-phase extraction at four time point in the interval of 5–6 h during 24-h. Shaded area was correspond to the dark condition. Experiments were based on triplicate biological replicates

To determine how orchid floral scent emission varied throughout the day, *P. violacea* plants were first transferred to a 16 h/8 h L/D condition on Dd and grown under this photoperiod for 5 days. The floral scent of *P. violacea* was then collected at a 5–6-h interval on D + 4. Monoterpene emission increased at 4:00 a.m. to 10:00 a.m. internal, reached a peak at 10:00 a.m. to 16:00 p.m. interval, decreased thereafter, and was undetectable at midnight (Fig. 1a, b). These results indicate a diurnal monoterpene emission of *P. violacea* flowers.

To investigate whether the floral monoterpene emission is regulated by an internal oscillator, plants were further treated with constant light or constant dark conditions over 4 days (D + 5 to D + 8). Previously, monoterpene biosynthesis is found to be developmentally regulated (Hsiao et al. 2008). Indeed, the monoterpene emission levels increased and peaked on D + 5 during daytime (Fig. 1a). Under constant light, although the periodical peaks were gradually decreased from D + 6 to D + 8, they did exhibit a regular and similar pattern during the rest of the photoperiod, indicating the monoterpene emission is under rhythmic regulation (Fig. 1a). In contrast, monoterpene emission decreased significantly as the plants were placed in the constant dark (Fig. 1b). A minor peak of monoterpene emission (approximately one tenth of the level in the constant light condition) was still observed at the 10:00 a.m. to 16:00 p.m. internal on D + 5 (Fig. 1b), and the emission shifted to the 4:00 a.m. to 10:00 a.m. interval on D + 6, and even shifted to the midnight interval on D + 7 and D + 8 (Fig. 1b). The above results indicate a circadian regulation of floral monoterpene emission, and the effect of light as well.

### Effects of various light qualities on monoterpene emission

**Fig. 2:**
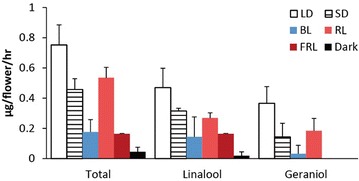
Emission of monoterpenes from *P. violacea* flowers in different light condition. The plants were treated with 100 μmol m^−2^ s^−1^ white light in long day (18 h/6 h L/D, LD) and short day (6 h/18 h L/D, SD) conditions, 270 μmol m^−2^ s^−1^ blue light (BL), 30 μmol m^−2^ s^−1^ red light (RL), 5 μmol m^−2^ s^−1^ far-red light (FRL), and darkness (dark). Scent was collected on D + 5 of an individual *P. violacea* flower. Experiments were based on triplicate biological replicates

To determine how the light quality could affect the orchid floral scent emission, we treated plants with various light wavelengths. Naturally, *P. violacea* is a native species in Malaysia and blooms in summer, and is considered as a long-day plant. Actually, the short-day condition (8 h/16 h L/D) reduced the floral monoterpene emission (Fig. 2). Therefore, we treated the orchids in the long-day condition (16 h/8 h L/D) with specified wavelengths, including the white light, blue light (270 μmol m^−2^ s^−1^), red light (30 μmol m^−2^ s^−1^), far-red light (5 μmol m^−2^ s^−1^) and darkness. The emission of linalool and geraniol was higher under white light than those under the other light spectra for *P. violacea* (Fig. 2). Among them, blue light, far-red light and dark had more effects than red light on the monoterpene emission. In contrast to linalool, the emission of geraniol nearly diminished under far-red-light and dark treatment in *P. violacea*.

**Fig. 3:**
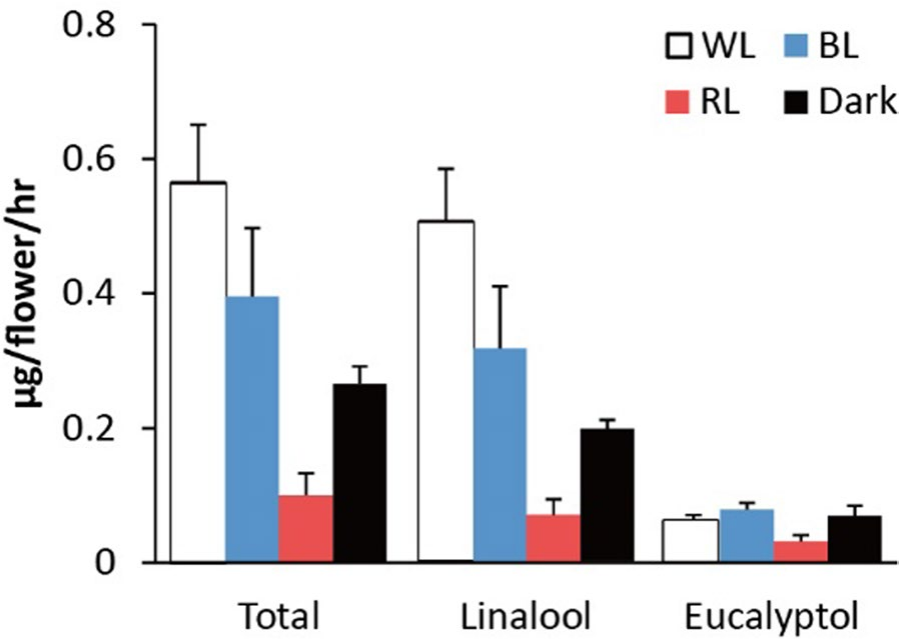
Emission of monoterpenes from *P.* I-Hsin Venus ‘KHM2212’ in different light conditions. The plants were treated with 50 μmol m^−2^ s^−1^ white (WL), red (RL), and blue (BL) light conditions, and darkness (dark). Scent was collected from six flowers on D + 5. Experiments were based on duplicate biological replicates

To see whether the offspring of *P. violacea* exhibits the similar response to different light treatment as their ancestor, we then analyzed the responses of two commercial *Phalaenopsis* cultivars containing *P. violacea* background to the light spectra. One is ‘KHM2212’ whose ancestry contains 15.63% of *P. violacea.* ‘KHM2212’ plants were basically with identical genetic background since they are micropropagated in tissue culture and their responses to different light were consistent (Fig. 3). ‘KHM2212’ emitted linalool and eucalyptol, and the emission of linalool was higher under white-light treatment than all the other light spectra (Fig. 3), similar to the observation in *P. violacea* (Fig. 2). The difference was that the red-light treatment reduced the emission of linalool more significantly than blue light and darkness in ‘KHM2212’ (Fig. 3). In addition, those various light spectra had little effect on the emissions of eucalyptol (Fig. 3), a compound not detected in the volatile profile of *P. violacea*. We deduced that the differences might be resulted from the genetic constitution of ‘KHM2212’ other than *P. violacea*.

**Fig. 4:**
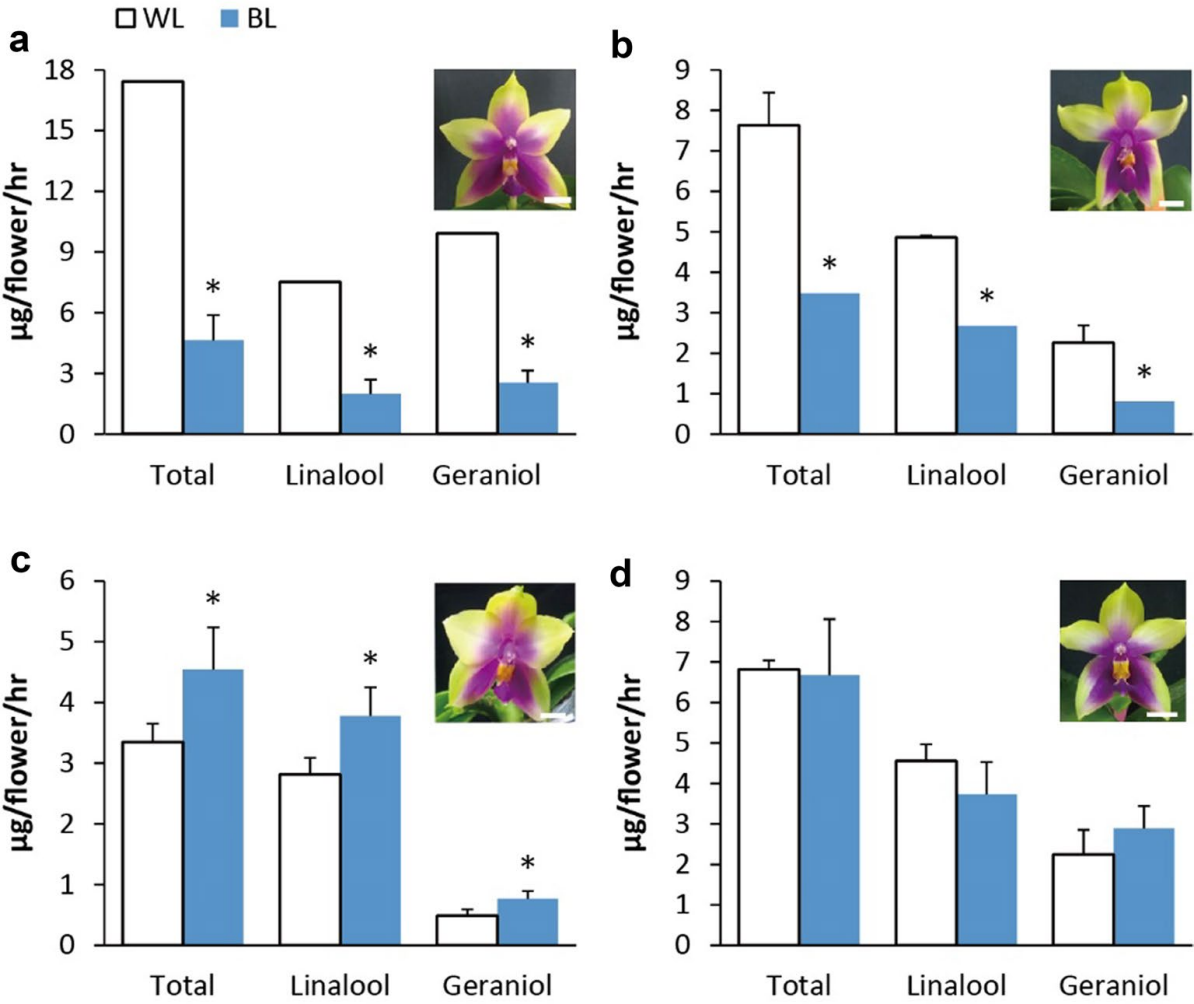
Emission of monoterpenes of four individual plants of *P.* Meidarland Bellina Age ‘LM128’. The plants were treated with 100 μmol m^−2^ s^−1^ white light (WL), and 270 μmol m^−2^ s^−1^ blue light (BL) conditions. The replicate number for each plants was listed as following: **a** No. 1 plant: *n* = 3 for white light (WL), and *n* = 4 for blue light (BL). **b** No. 2 plant: *n* = 1 for WL, and *n* = 4 for BL. **c** No. 3 plant: *n* = 3 for both WL and BL. **d** No. 4 plant: *n* = 3 for WL, and n = 1 for BL. Different letters on the column indicate significant difference by Student’s *t* test (*p* value < 0.1). Scale bar = 1 cm

Another *Phalaenopsis* hybrid examined was ‘LM128’ containing the ancestry of *P. bellina* (50%) and *P. violacea* (24.22%). ‘LM128’ is an orchid hybrid generated from at least seven generations of breeding and six native species were involved, leading to its complicated genetic background. We selected four ‘LM128’ seedlings plants derived from independent lines and found that the floral scent emission for each individual plant responded differentially to white-light and blue-light conditions (Fig. 4). Among them, two plants showed similar responses to both light spectra as *P. violacea*, in which blue-light treatment reduced the scent emission (Fig. 4a, b). The other two responded differently, one seedling emitted higher monoterpene under blue light than white light (Fig. 4c) and the other showed the same level of emission (Fig. 4d). Although the responses were different among the four plants, the reactions of linalool and geraniol emission to both light spectra were consistent in each ‘LM128’ individual (Fig. 4).

Together, these results imply that whole spectra of light (i.e. white light) is conferred the optimal emission of scent compounds in most *Phalaenopsis* orchids with *P. violacea* background.

### Analysis of the upstream regulatory fragments of structural genes and TFs for monoterpene biosynthesis

**Fig. 5:**
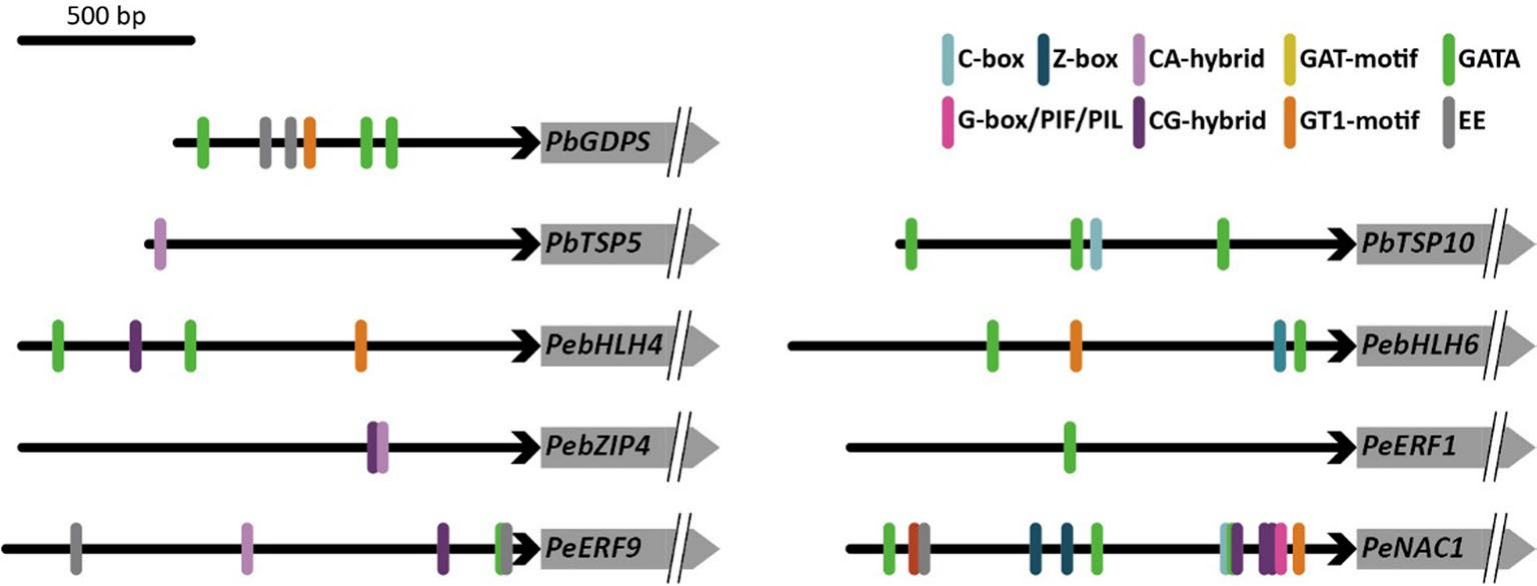
Light- and circadian-responsive elements on the upstream regulatory fragments of three structural genes and six transcription factors for floral monoterpene biosynthesis in *P. bellina*. The distinct *cis*-elements on each fragments were represented by their location predicted by PlantPAN (Chow et al. 2015). The gene length was not shown in proportion to actual length. Scale bar of upstream regulatory fragments = 500 bp

In the light signaling pathway, HY5 binds to the promoters of early light-responsive genes and TF genes to regulate plant development and photomorphogenesis (Lee et al. 2007). We examined whether the upstream promoters of the structural genes and TFs for monoterpene biosynthesis in *Phalaenopsis* contain any HY5-interacting ACGT-containing elements (ACEs), such as C-box (GACGTC), Z-box (ATACGGT), CA-hybrid (GACGTA), CG-hybrid (GACGTG), as well as G-box (CACGTG) for PIF recognition (Lee et al. 2007; Zhang et al. 2011). Among the genes analyzed, *PbNAC1* contained a large number of ACE motifs (*n* = 7), implying that it might be a target of HY5 protein (Table 1, Fig. 5). In addition, several other *cis*-acting elements related to light and circadian clock signaling were also identified in the 1.5-kb promoter of *PbNAC1* by using PlantPAN (Chow et al. 2015). These included GATA-box (Luo et al. 2010), GT1-motif (Zhou 1999), and evening elements (EE) for CCA1 and LHY interaction (Alabadí et al. 2001) (Table 1, Fig. 5). These *cis*-elements were identified in the promoter of structural genes and TFs for monoterpene biosynthesis as well, although in a smaller number (Table 1, Fig. 5). *PbGDPS*, a key enzyme in the monoterpene biosynthesis (Hsiao et al. 2008), was the only one structural gene containing EEs, suggesting that it might be a downstream gene of circadian clock pathway.

**Table 1:** c*is*-elements on the promoter fragments of scent-related structural genes and TFs

Gene name	Promoter length	HY5 interaction					Light-responsive		Circadian
		C-box^a^	Z-box	G-box^a^	CA	CG	GATA	GT1-motif	EE
					Hybrid	Hybrid			
*PbGDPS*p	1077	–^b^	–	–	–	–	3	1	2
*PbTPS5*p	1161	–	–	–	–	–	–	–	–
*PbTPS10p*	1506	1	–	–	–	–	3	–	–
*PebHLH4*p	1538	–	–	–	–	1	2	1	–
*PebHLH6*p	1673	–	–	1	–	–	2	1	–
*PebZIP4*p	1537	–	–	–	1	1	–	–	–
*PeERF1*p	1500	–	–	–	–	–	1	–	–
*PeERF9*p	1565	–	–	–	1	1	–	–	2
*PeNAC1*p	1501	1	2	1	–	3	3	2	1

^a^The number of palindromic sequences was counted by the sense strand

^b^The dash indicates that the *cis*-element is not detected

### Identification of factors involved in light and circadian clock signaling pathways

**Fig. 6:**
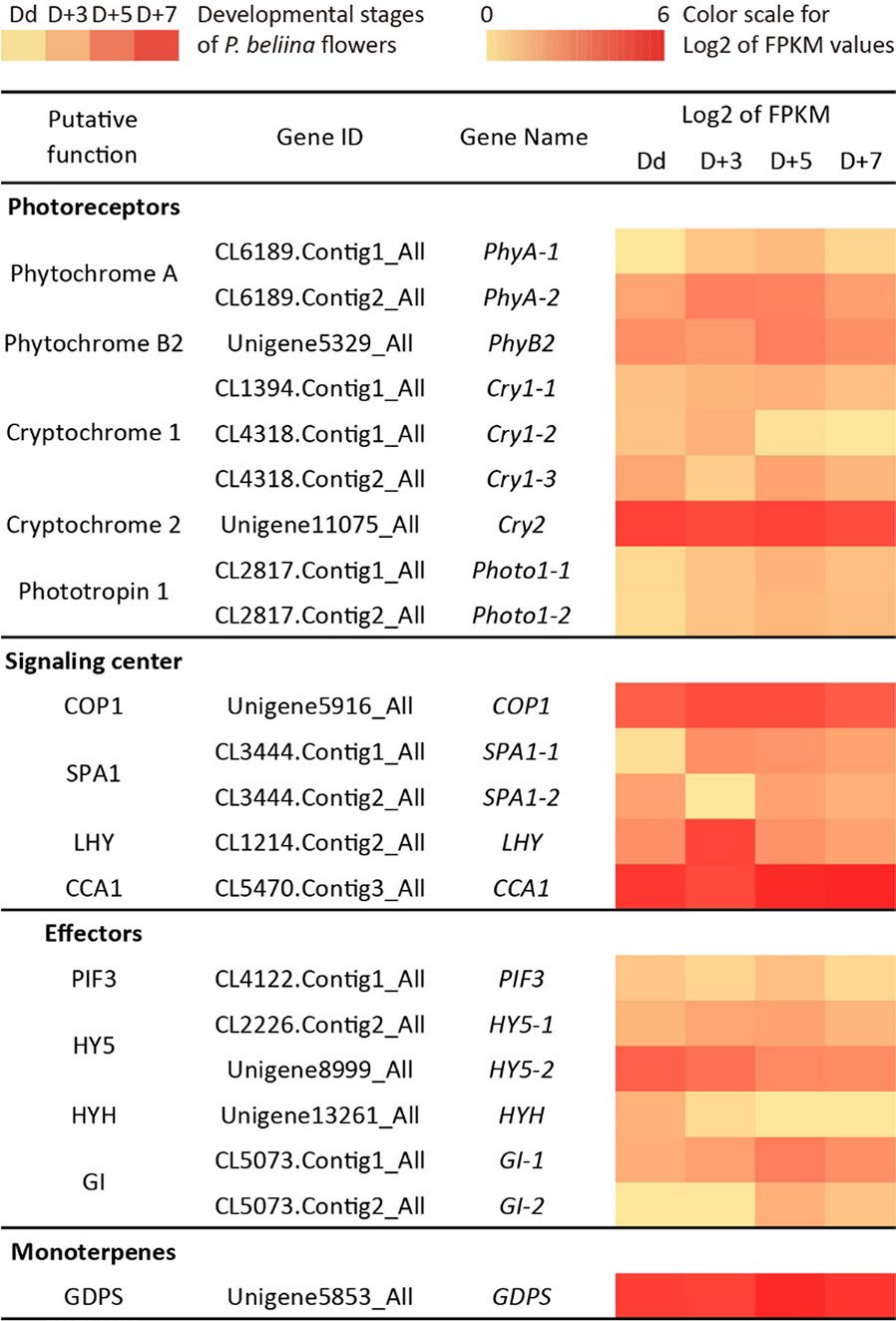
Expression profiles of putative genes encoding components involved in light and circadian clock signaling pathway in *P. bellina* floral transcriptomes. The gene expression levels are represented by a color gradient from red to light-red

Both light and circadian signaling pathways were well studied in *Arabidopsis* (Dodd et al. 2015; Lau and Deng 2012). We identified their homologous genes in *P. bellina* floral transcriptome to explore the candidate factors responsible for the circadian- and light-regulating scent emission in *Phalaenopsis* flowers. The transcriptomic data were constructed from four floral developmental stages, including Dd, D + 3, D + 5 and D + 7 (Chuang et al. unpublished). The expression levels of the homologous genes were determined by log2 of FPKM values, and compared to that of *PbGDPS* (Fig. 6). The putative genes encoding the factors involved in light and circadian signaling pathways were successfully isolated in *P. bellina* transcriptome, and several ones showed higher expression, including putative genes encoding PHYTOCHROME A, PHYTOCHROME B, CRYPTOCHROME 2, COP1, LHY, CCA1, HY5 and GI (Fig. 6). As we identified the *cis*-elements related to the light and circadian responses in the structural genes and TFs for monoterpene biosynthesis, it is worth to study the function of these putative genes in *Phalaenopsis* orchids in the future.

## Discussion

### Light and/or circadian rhythm regulation on the emission of monoterpenes

*Phalaenopsis bellina*, or *P. violacea*, emits monoterpenes during daytime and is considered as day-pollinated flowers. Here, we present that this diurnal emission is affected mainly by circadian clock and to a lesser degree by the light factor. This is different from other plant species, whose diurnal rhythm is usually regulated by a single factor. *Rosa hybrida* L. cv. Honesty, *Antirrhinum majus*, and *Rosa damascena semperflorens* emit monoterpenes regulated by a circadian clock regardless of the light stimulus (Helsper et al. 1998; Dudareva et al. 2003; Picone et al. 2004). In contrast, in *Mahonia japonica* and *Rosa hybrida* cv. Fragrant Cloud, the diurnal emission of monoterpenes is determined primarily by the photoperiod (Picone et al. 2002; Hendel-Rahmanim et al. 2007).

The monoterpene emission patterns are likely to be parallel the expression of structural genes. In *A. majus* flowers, the terpene backbone biosynthesis pathway, *GDPS* and monoterpene synthase are all under circadian regulation (Dudareva et al. 2003, 2005; Tholl et al. 2004). The emission of monoterpenes in *P. bellina* matches the expression patterns of *PbGDPS* during flower development (Hsiao et al. 2008). Further study of whether these structural genes contribute to the diurnal emission will help to clarify how monoterpene biosynthesis is controlled in a day.

### HY5 integrates the light and circadian signaling pathway for terpene biosynthesis

HY5, a specialized basic leucine zipper (bZIP) TF, plays an important role in mastering both light and circadian signaling pathway (Gangappa and Botto 2016). The role of HY5 on terpene biosynthesis is recently unraveled. *Arabidopsis* responds to illumination by integrating HY5 to repress the mevalonate (MVA) pathway and activate methylerythritol phosphate (MEP) pathway to increase plastidial terpenes for photosynthesis (Rodríguez-Concepción et al. 2004). As grape berries are shaded from light (sunlight exclusion), the downregulation of the terpene biosynthesis leads to the reduction of terpene content and altered aroma (Zhang et al. 2014, 2017). HY5 might be involved in the light-induced terpene production in grapes (Costantini et al. 2017). Moreover, HY5-binding sites are identified in the promoter regions of terpene biosynthesis genes (Lee et al. 2007). In *Artemisia annua, a* circadian-regulated β-pinene synthase gene, *QH6*, contains G-box motif to interact with HY5 for retaining rhythmic expression under constant condition (Zhou et al. 2015). Other regulator in light signaling pathway, such as PIF5, is involved in terpene biosynthesis as well (Mannen et al. 2014). Here, we identified several HY5-interacting motifs on the upstream regulatory fragments of structural genes and TFs, especially PbNAC1, suggesting that the light and circadian clock signals might manage monoterpene biosynthesis in *P. bellina* by controlling these genes.

Naturally, the striking changes of light at dawn and dusk help to entrain the circadian clock. Phytochrome is responsible for the red-light input and cryptochrome for the blue-light (Devlin and Kay 2000). Our results show that *P. violacea* responded differently to various light qualities. It is intriguing to know how the signaling components isolated in this study work together to regulate monoterpene biosynthesis in *Phalaenopsis* orchids, including photoreceptors and COP1.

The floral scent of *P. bellina* is composed by monoterpenes and its biosynthesis is regulated by multiple factors. *PbGDPS* is expressed in the epidermal cells on the surface of perianth (Hsiao et al. 2008), suggesting a factor for its tissue specificity. Moreover, the development factor is involved to induce the scent biosynthesis at full-bloomed stages (Chuang et al. unpublished). In this study, we present that both circadian clock and light factor are required for the diurnal emission. These factors cooperate to establish the optimal spatial and temporal emission patterns of monoterpenes in *Phalaenopsis* flowers.

## Conclusion

The present study indicates that the diurnal emission of monoterpenes from *P. violacea* flowers is controlled by a circadian clock and light factors. In addition, white light was the most optimal light condition to emit monoterpenes in four *Phalaenopsis* orchids examined. The light and circadian signal might be associate with floral monoterpene emission by regulating the transcription factors for monoterpene biosynthesis.
